# Effect of Acupuncture Intervention and Manipulation Types on Poststroke Dysarthria: A Systematic Review and Meta-Analysis

**DOI:** 10.1155/2020/4981945

**Published:** 2020-09-14

**Authors:** Young-Jae Park, Jin-Moo Lee

**Affiliations:** ^1^Department of Biofunctional Medicine and Diagnostics, College of Korean Medicine, Kyung Hee University, Seoul, Republic of Korea; ^2^Department of Diagnosis and Biofunctional Medicine, Kyung Hee University Hospital at Gangdong, Seoul, Republic of Korea; ^3^Department of Gynecology, College of Korean Medicine, Kyung Hee University, Seoul, Republic of Korea; ^4^Department of Women Health Clinic, Kyung Hee University Hospital at Gangdong, Seoul, Republic of Korea

## Abstract

This study aimed to evaluate the effect of acupuncture intervention and manipulation types on poststroke dysarthria. Electronic database, including PubMed, CENTRAL, Scopus, RISS, and CNKI, were searched for randomized controlled trials (RCT), treating dysarthria using acupuncture, speech-language therapy (SLT), and general management (GM), published before April 2019. The number, distribution, intensity, depth, and repetition of acupuncture and bleeding therapy on the sublingual veins were considered as manipulation types. Risk of bias of the included trials was evaluated, and their efficacy was assessed using risk ratio (RR) and the standard mean differences in the Frenchay Dysarthria Assessment and Speech Function Grading, with 95% confidence intervals (CIs).Fifteen RCT trials involving 1453 patients were isolated. Electroacupuncture plus SLT and manual acupuncture plus SLT were more effective than SLT only, respectively (RR = 1.520, 95% CI [1.183–1.952], RR = 1.380, 95% CI [1.281–1.488]). The clinical efficacy of acupuncture plus GM was higher than that of GM alone (RR = 1.165, 95% CI [1.050–1.293]). Meta-ANOVA showed that none of the manipulation types increased the clinical efficacy of acupuncture on dysarthria. The methodological quality was low. In conclusion, our study suggests that the effect of acupuncture on poststroke dysarthria may be maximized when manual acupuncture or electroacupuncture is combined with SLT, irrespective of manipulation types.

## 1. Introduction

Stroke is the second leading cause of death, following heart disease, with 15 million people suffering strokes annually [1]. The 5 million survivors suffer various physical and mental disabilities, including dysarthria: a condition of speech impairment involving articulation, phonation, nasality, intelligibility, and efficiency [2]. Reportedly, 20–22% of patients suffer dysarthria after stroke [3, 4]. Moreover, dysarthria impairs patients' self-identity, social relationships, education, and quality of life [5]. Speech and language therapy (SLT)—including strategies to improve the strength of oral and vocal muscular movements, as well as respiratory support, phonation, and resonance—is primarily considered to treat dysarthria [2].

Acupuncture treatment (AT) has been widely used for poststroke rehabilitation, and many systematic review and meta-analysis studies have addressed the effects of acupuncture on poststroke complications such as shoulder pain [6], spasticity [7], depression [8], insomnia [9], and dysphagia [10]. Previously, Chen et al.'s study, written in Chinese, showed that the clinical efficacy of AT combined with SLT was higher than that of the SLT-only group [11]. However, Chen's study did not limit the control group to general management or SLT and included herbs or multiple acupuncture interventions in the treatment group, which may have confounded the pure effect of AT on dysarthria with the effect of other interventions.

Together with the acupuncture intervention type, it is generally accepted that some acupuncture manipulation skills, including intensity, depth, repetition, and distribution of needling, and bleeding therapy using needle pricking affect the clinical efficacy of AT [12]. However, a few studies have addressed the efficacy of AT manipulation skills on dysarthria, using a systematic meta-analysis. Therefore, this study aimed to estimate the pure effect of AT on poststroke dysarthria by the intervention type and to examine which types of AT manipulation may be effective on the poststroke dysarthria.

## 2. Methods

### 2.1. Search Strategy

The following databases were searched for articles (with no language restrictions) related to acupuncture, dysarthria, and stroke that were published before April 2019: the Cochrane Central Register of Controlled Trials, PubMed, Scopus, National Digital Science Library, Research Information Sharing Service, Korea Studies Information Service, and China National Knowledge Infrastructure. The search strategy followed the Preferred Reporting Items for Systematic Reviews and Meta-Analyses (PRISMA) guidelines, without a date limitation [13]. The search keywords used were dysarthria, acupuncture, acupuncture therapy, acupoint, electroacupuncture, auricular acupuncture, ear acupuncture, pharmacopuncture, randomized controlled trial, random allocation, clinical trial, placebo, stroke, apoplexy, brain infarction, cerebral infarction, and cerebral hemorrhage. These terms were used as free-text terms to search the Korean-language and Chinese-language databases, whereas free-text terms and MeSH terms were both used to search the English-language databases.

### 2.2. Data Extraction

Two reviewers independently extracted data from the retrieved articles using a predefined template that included the author name, publication year, sample size, average age, time since onset, intervention types, methods of intervention including AT, SLT, and general management (GM), primary outcome, secondary outcomes, study results, characteristics of acupuncture manipulation, and adverse effects. When the reviewers disagreed, a consensus was reached through discussion.

### 2.3. Types of Subjects and Interventions

The trials involving adults over 18 years of age diagnosed as having had a stroke were eligible; there was no restriction on time since the onset of the stroke. In terms of intervention, only randomized controlled trials (RCT) were considered. To this end, we limited the intervention types to two categories: AT plus GM versus GM and AT plus SLT versus SLT only. In this study, GM was restricted to the treatments used to control patients' vital signs and glucose levels. The studies which documented intravenous injection of herbs were excluded. There was no restriction on the acupuncture type, and therefore, not only manual acupuncture but also electroacupuncture (EA), auricular acupuncture (AA), and pharmacopuncture (PA) were considered. The trials which simultaneously used two or three AT interventions were excluded. EA and PA trials were retrieved only when the acupoints used in EA or PA treatment were identical to manual AT. If there were differences in acupoints selection between manual AT, EA, or PA, it was regarded as AT plus EA or AT plus PA; thus, they were excluded. A study where acupoints were variable according to pathological patterns was also excluded. With regards to SLT, trials were considered eligible for this study if they (1) trained the patients in breathing, relaxation, articulation, and movement of the tongue, face, and lips for at least 20 minutes per session and (2) involved a total SLT treatment time of that was equal to the acupuncture treatment time.

### 2.4. Types of Acupuncture Manipulations

A total of six manipulation types from the retrieved studies were considered: total needle numbers, distribution, intensity, depth, and repetition of needling, as well as bleeding of the tongue induced by needle pricking. Trials that used fewer needles than the mean value of the retrieved trials were assigned to the “small number of needles group,” whereas those using more needles than the mean value were assigned to the “large number of needles group.” Trials that combined the acupoints of the upper and lower limbs and trunk with those of the tongue, face, head, or neck were assigned to the “broad distribution group,” whereas those that were limited to the acupoints of the tongue, face, head, or neck were assigned to the “local distribution group.” Trials that included bleeding of the tongue were assigned to the “bleeding group,” whereas those that involved no bleeding of the tongue were assigned to the “nonbleeding group.” Trials that documented whirling and thrusting methods of needling were assigned to the “strong-intensity group,” whereas those that documented no such methods were assigned to the “weak-intensity group.” Trials that inserted the needles to a depth of over 3 cm were assigned to the “deep insertion group,” whereas those that inserted needles to a depth of less than 3 cm were assigned to the “superficial insertion group.” Finally, trials were assigned to the “repetition group,” which repeated a whirling and thrusting manipulation two or three times at 10-minute intervals after the initial insertion, whereas those that conducted no such repetition were assigned to the “nonrepetition group.”

### 2.5. Outcome Measurement

Total scores and the *a-*points of the Frenchay Dysarthria Assessment (FDA) [14], as well as the Speech Function Grading (SFG) [15], were extracted from the retrieved articles. The FDA assesses the reflex, respiration, lips, jaw, soft palate, laryngeal, tongue, and intelligence categories of movements, rating 28 items on a 5-point scale ranging from “*a*” (intact movements) to “*e*” (inability to perform any movements) [14]. The *a*-point items are totaled, and the rating score of the FDA ranges from 0 to 28 points. The score is, then, transformed into five grades as follows: ≥27 points, normal; 18–26 points, mild dysarthria; 14–17 points, moderate dysarthria; 7–13 points, severe dysarthria; and ≤6 points, highly severe dysarthria [16]. After that, changes in the FDA grading were transformed into four grades: completely improved, prominently improved, somewhat improved, and not improved. For example, “prominently improved” corresponds to an improvement by two grades in the FDA rating, whereas “somewhat improved” corresponds to an improvement by one grade [8, 16]. The SFG estimates patients' dysarthria by assessing the fluency of speech, the voice volume, articulation clarity, and ability to communicate normally. Like the four grades of the FDA, changes in the SFG are graded on a four-point scale. A change of two or three SFG grades indicates a prominent improvement, whereas a change of one grade corresponds to a slight improvement [15, 16]. The clinical efficacy estimated by changes in the FDA and SFG ratings was transformed into a dichotomous variable: complete, prominent, and slight improvements were denoted as “events,” whereas nonimprovements were denoted as “nonevents,” as in previous studies [1, 8].

### 2.6. Quality Assessment

The methodological risk and risk of bias of the studies were independently assessed by two reviewers, according to the checklist of the Cochrane Handbook for Systematic Reviews of Interventions [17]. This checklist consists of eight categories: (1) random sequence generation, (2) allocation concealment, (3–5) blinding of patients, personnel, and outcome assessors, (6) incomplete outcome data, (7) selective outcome reporting, and (8) other sources of bias. The risk of bias for each category was determined as “high,” “low,” or “uncertain,” and disagreements were resolved through discussion between the two reviewers.

### 2.7. Statistical Analysis

In the present study, the primary outcomes were improvement or nonimprovement, according to the four grading changes in the FDA and the SFG. Additionally, the total scores and numbers of improved *a*-point items of the FDA were the secondary outcomes. The risk ratio (RR) and 95% confidence interval (CI) of the retrieved studies were calculated to examine the differences in the primary outcomes. Hedges' *g*-values and standard mean differences (SMD) adjusting for bias due to sample numbers were calculated to examine the differences in the secondary outcomes [18]. Meta-ANOVA tests were conducted to examine whether there were any differences in clinical efficacy between intervention and manipulation types. As the homogeneity of sampling and study design between the retrieved studies could not be guaranteed, a random-effects model was considered [19]. *I*^2^ levels were examined to assess what proportion of overall variation was due to between-study heterogeneity [20]. Publication bias was examined using a funnel diagram and Egger's regression test [21]. If there was any publication bias, the trim-and-fill method was followed to examine the extent of the bias [22]. All statistical analyses were conducted using the “meta” R package [23]. In the meta-ANOVA and Egger's regression analyses, *P* values <0.05 were regarded as significant. *I*^2^ values >50% indicated significant heterogeneity.

## 3. Results

### 3.1. Study Description

**Figure 1:**
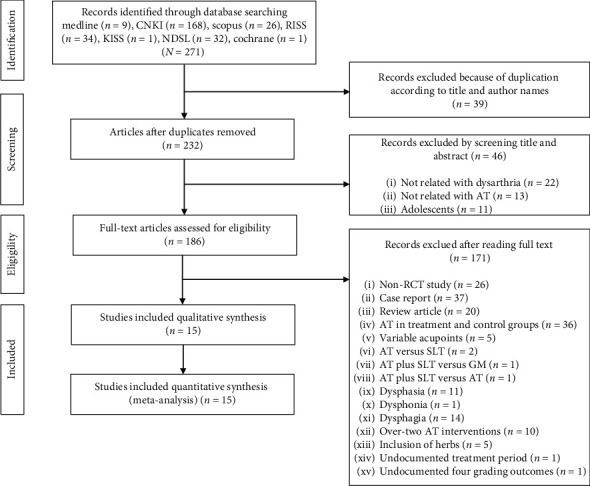
Title: Preferred reporting Items for Systematic Reviews and Meta-Analysis (PRISMA): flow diagram of identification, screening, eligibility, and inclusion process. Caption: AT, acupuncture treatment; GM, general management; RCT, randomized controlled trial; and SLT, speech-language therapy.

The initial search identified 271 potential studies (Figure 1). Among these, 15RCTs met the eligibility criteria (Table 1) [24–38]. All 15 articles were published in China and were written in the Chinese language; they included a total of 1453 patients. Articles retrieved from English-language databases were duplicates of those published in Chinese. The articles written in Korean were either not RCTs or did not include AT. In ten articles, patients were diagnosed as having experienced a stroke using computed tomography or magnetic resonance imaging [24–26, 29–34, 37]. In five articles, stroke was diagnosed according to clinical criteria [27, 28, 35, 36, 38]. Table 1 summarizes the subject's age, time since onset, characteristics of acupuncture and SLT interventions, outcome results, and manipulation types. Eleven of the articles considered a change in the FDA grading as the primary outcome [25, 26, 28–32, 34], whereas the other four considered the change in the SFG grading [24, 27, 33, 35]. Twelve articles entailed an intervention design of AT plus SLT versus SLT only [24, 26–28, 30–36, 38], whereas two involved AT plus GM versus GM [25, 29], and one involved EA plus SLT versus SLT [37]. None of the studies compared AA plus SLT or PA plus SLT with SLT only or GM only. All 15 articles documented the six manipulation types. However, only 10 articles documented time since onset [24, 26, 27, 29, 32–35, 37, 38].

### 3.2. Risk of Bias

Table 2 summarizes the risk of bias in the 15 articles. Although all the articles reported adequate randomization, only five addressed the methods of randomization (random number table or computerized random number generator) [25, 28, 32–34]. Allocation concealment and blinding of the outcome assessor were only addressed in two articles [32, 35]. Six articles reported incomplete outcome data [28, 31–33, 35, 36]. The risk of bias related to the blinding of patients and personnel to the intervention and the risk of bias of other problems were uncertain in all 15 articles.

### 3.3. Main Findings

#### 3.3.1. Effects of Acupuncture on Dysarthria, according to Outcome Measurements

**Figure 2:**
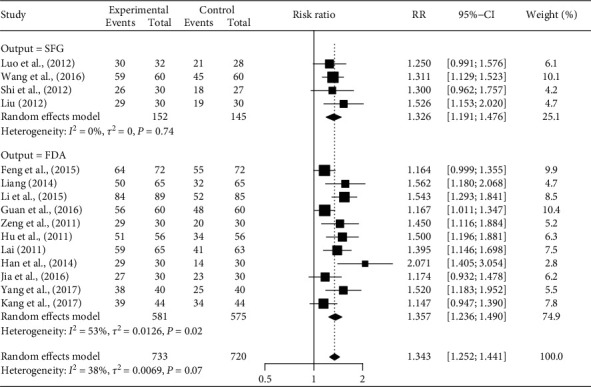
Title: forest plot of the efficacy of acupuncture on dysarthria by SFG and FDA scores. Caption: CI, confidence interval; FDA, Frenchay dysarthria assessment; RR, risk ratio; SFG, speech function grading.

Figure 2 depicts the effects of AT on dysarthria, by SFG and FDA outcomes. In the four studies that examined the clinical efficacy of AT using an SFG score [24, 27, 33, 35], AT plus SLT or AT only was more effective than SLT only or GM only (RR = 1.326, 95% CI [1.191–1.476]). In the eleven studies that examined the clinical efficacy of AT using an FDA score [25, 26, 28–32, 34], AT plus SLT or AT plus GM, or EA plus SLT, were more effective than SLT only or GM only (RR = 1.357, 95% CI [1.236–1.490]). Meta-ANOVA showed no difference in the RR between the SFG and FDA scores (*Q*_(1)_ = 0.11, *P*=0.745).

**Figure 3:**
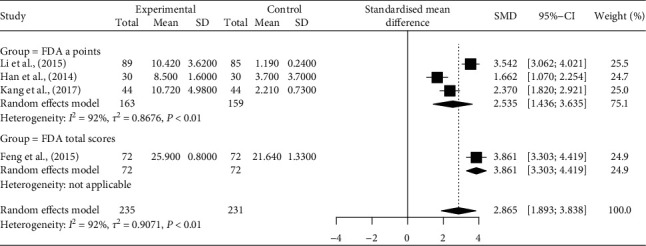
Title: forest plot of the efficacy of acupuncture on dysarthria by changes in FDA a-points and total scores. Caption: CI, confidence interval; FDA, Frenchay dysarthria assessment; SD, standard deviation; SFG, speech function grading; SMD, standardized mean difference.

Figure 3 depicts the effects of AT on dysarthria, by FDA-*a*-points and total scores. In the three studies that examined the changes in FDA-*a-*points [28, 34, 38], AT plus SLT was more effective than SLT only (Hedges' *g* = 2.535, 95% CI = 1.436–3.635). In one study that examined the changes in the FDA total score [25], AT plus GM was more effective than GM only (Hedges' *g* = 3.861, 95% CI [3.303–4.419]). There was not any significant heterogeneity between the RRs of SFG and FDA ratings (*I*^2^ = 38%, *P*=0.07). However, the studies that evaluated clinical efficacy using FDA-*a*-points showed significant heterogeneity (*I*^2^ = 92%, *P* < 0.01), and these studies also showed significant heterogeneity with the study using FDA total scores (*I*^2^ = 92%, *P* < 0.01).

#### 3.3.2. Effects of Acupuncture Intervention Types on Dysarthria

**Figure 4:**
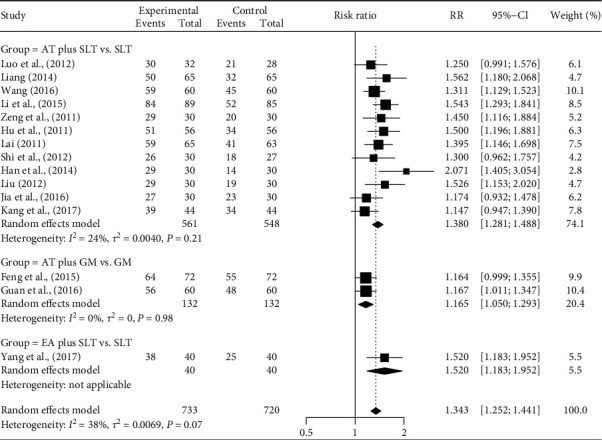
Title: forest plot of the efficacy in dysarthria by acupuncture intervention types. Caption: AT, acupuncture treatment; CI, confidence interval; EA, electroacupuncture; GM, general management; RR, risk ratio; SLT, speech-language therapy.

Figure 4 depicts the effects of AT on dysarthria, by AT intervention types. In twelve studies, manual AT plus SLT was more effective than SLT only (RR = 1.380, 95% CI [1.281–1.488]). In two studies [25, 29], manual AT plus GM was more effective than GM only (RR = 1.165, 95% CI [1.050–1.293]). In one study [37], EA plus SLT was more effective than SLT only (RR = 1.520, 95% CI [1.183–1.952]). The meta-ANOVA test showed that there were between-intervention type effects (*Q*_(2)_ = 8.04, *P*=0.018). There was little heterogeneity among the three intervention types (*I*^2^ = 38%, *P*=0.070).

#### 3.3.3. Effects of Acupuncture Manipulation Types on Dysarthria

As the mean number of needles used was 9.2, studies that used nine or fewer needles were assigned to the “small number group,” whereas those that used ten or more were assigned to the “large number group.” Table 3 summarizes the meta-ANOVA results of the six acupuncture manipulation types (number and distribution of needles, intensity, depth, and repetition of needling and induced bleeding of tongue substance and sublingual veins; HN-EX12, HN-EX13 points). None of the six manipulation types showed any differences in the clinical efficacy on poststroke dysarthria.

### 3.4. Publication Bias

**Figure 5:**
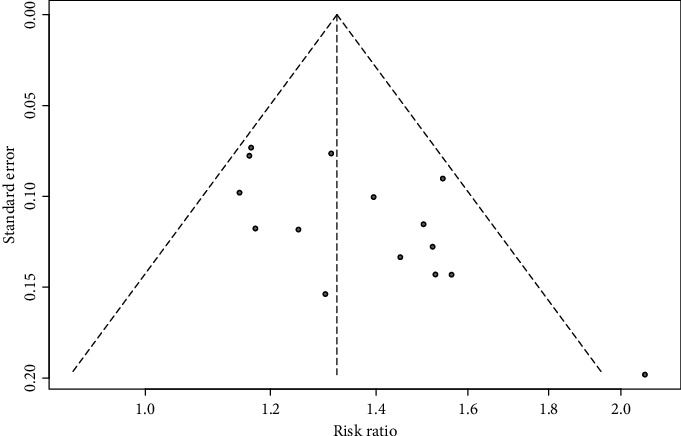
Title: a funnel plot of the effect size of Frenchay dysarthria assessment and speech function grading changes between the acupuncture and control groups.

Figure 5 depicts funnel plots of the 15 retrieved studies. Egger's regression test showed significant publication bias (*t* = 2.907, degrees of freedom: 13, *P*=0.012). The trim-and-fill method suggested the addition of five trials to the original study pool, and the estimated RR of the 20 trials was 1.264 (95% CI [1.171–1.365]).

### 3.5. Adverse Effects

Among the 15 trials included, two reported pain at the acupuncture sites [24, 34]. None of trials reported any adverse effects due to invasive acupuncture manipulations, such as strong stimulation or deep insertion.

## 4. Discussion

In this systematic review and meta-analysis, we limited the control groups to SLT only or GM only, which allowed comparison of the effect size of AT plus SLT with that of SLT only and to compare the effect of AT plus GM with that of GM only. As mentioned above, a previous study included herb treatments in the control group and multiple acupuncture intervention types in the treatment group [11]. Moreover, the previous study used a fixed-effect model, although the retrieved studies could not guarantee the homogeneity of the study process [11]. To overcome these limitations, we included the RCTs which used one AT intervention type as the treatment group and restricted the control group to the SLT-only or GM-only group. After that, we estimated the efficacy using a random effect model.

One main finding of our study is that the clinical efficacy of AT on dysarthria differed by the AT intervention type. Among the three intervention types, the effect size of EA plus SLT was the highest, and that of manual AT plus SLT was higher than that of manual AT plus GM. However, the retrieved number of EA plus SLT studies was small, and more clinical evidence that the effect of EA plus SLT may be higher than that of SLT only is needed. In this study, we transformed four grading outcomes to dichotomous variables according to the previous systematic review studies [1, 8]. However, this transformation has the possibility of overestimation of successful outcomes because among the four grades, three grades were attributed to “event,” and only one grade was attributed to “nonevent.” Instead, Hedges' *g* value is known to minimize the bias due to the transformation or small sample size [18]. At the very least, our study results suggest that the clinical efficacy of AT plus SLT or AT plus GM is higher than that of SLT only or GM only when minimizing effect size bias using Hedge' *g* value.

Interestingly, the effect size of AT plus GM or AT plus SLT did not differ by the six manipulation types. As mentioned above, acupuncture is not a single technique but rather the sum of various considerations, including intensity, depth, and numbers and distribution of needles [12]. Although all the retrieved studies used these manipulation skills in order to maximize the effect of AT intervention, it seems that the manipulation skills did not increase the effect of AT intervention on dysarthria. It is uncertain why numerous, broad, strong, repetitive, and deep insertion did not increase the clinical efficacy of AT on poststroke dysarthria. Under nonpainful conditions, acupuncture stimuli are projected to efferent autonomic nerves via the somatoautonomic reflex pathway, without strong or deep insertion [39]. Considering that dysarthria is categorized as a nonpainful consequence of stroke, one possibility is that acupuncture for the treatment of poststroke dysarthria may not require strong, deep, and repetitive stimuli because activation of the somatoautonomic reflex may not need strong and deep stimuli to facilitate recovery of dysarthria.

Regarding the effect of bleeding therapy, some animal studies reported that bleeding therapy on the peripheral regions of the legs was effective for the ischemic recovery via activation of blood circulation [40, 41]. Our study results showed that there were not any differences in clinical efficacy between the bleeding and nonbleeding groups. For this reason, one possibility is that bleeding therapy is beneficial mainly for the early stage, that is, within 72 hours after stroke [40, 41]. As almost all patients retrieved in our study were not categorized into the early-stage patients, bleeding manipulation using AT pricking may not have increased clinical efficacy. However, the number of RCTs using broad distribution, weak stimulation, and nonbleeding manipulations was small, and thus, more clinical evidence for the manipulation skills is needed.

Regarding publication bias, Egger's regression test showed a significant publication bias. However, the results of the trim-and-fill method recommended the addition of five trials to overcome publication bias and predicted a decrease in the effect size of 8%. Therefore, it seems that the publication bias in our study, if present, was not significant. When examining the risk of bias, only five studies documented adequate randomization methods, although another ten used the term randomization. Only one article addressed allocation concealment, and none of the studies documented blinding of the patients and personnel to the intervention, which indicates that the methodological quality of the reviewed studies was low. In terms of adverse events, two studies reported pain at the acupuncture sites, and no other adverse events were documented. Therefore, it seems that AT interventions may be safe for patients with poststroke dysarthria, irrespective of manipulation factors such as intensity, depth, and repetition of needling.

The present study has some limitations. The number of RCTs of AT plus GM and EA plus SLT was small. This study could not compare other acupuncture types, including PA and AA, with SLT or GM. As none of the retrieved studies reported the clinical efficacy according to the stroke type (e.g., cerebral infarction and hemorrhage), or according to lesion type, it is challenging to examine whether clinical efficacy of AT intervention types may differ, according to the initial stroke type. Publication bias and low methodological quality of the studies should be overcome. The retrieved trials reported only the short-term or mid-term effects of AT plus SLT or AT. Thus, the long-term effects of AT intervention on poststroke dysarthria need more research. Further studies are required to address these limitations.

## 5. Conclusions

This systematic review and meta-analysis involving 1453 patients suggests that the effect of acupuncture on poststroke dysarthria may be maximized when manual AT and EA are combined with SLT. Manipulation skills such as strong, deep, broad, and repetitive insertion and bleeding on the tongue substance may not increase the effect of AT on dysarthria. Further studies are needed to overcome the limitations of the small number of RCTs, lack of data on long-term effects, publication bias, and low methodological quality.

## Figures and Tables

**Table 1:** Characteristics of randomized controlled trials of acupuncture treatments for dysarthria.

Author (year)	Number of cases (men/woman)	Average age or range of age (years)	Time since onset (days)	Regime (a)	Regime (b)	Outcome measurement	Results	Adverse events	Manipulation of acupuncture					
									Total no.	Location	Bleeding	Intensity	Depth	Repetition
Zeng (2011)	(a) 30 (18/12) (b) 30 (19/11)	(a) 60.5 (b) 62.7	Undocumented	(1). AT (PC6, GV26, SP6, GB20, GB12, TE17, bleeding AT of the EX-HN12 and EX-HN13; 5 sessions a week for 4 weeks). (2). The same regime using SLT in the control group (b)	(1). SLT (passive tongue and lip movement (severe), active tongue and lip movement training, articulation, and speech speed training (moderate); a total of 30 sessions)	FDA	Significant differences in effective rates (*P* < 0.01)	None	13	Broad	+	Strong	Deep	—
Hu (2011)	(a) 56; (b) 56	40–75	Undocumented	(1). AT (GV20, CV23, and two para-CV23 points, bleeding AT of the EX-HN12 and EX-HN13, 5 sessions a week for 4 weeks, 30 min. per session). (2). The same regime using SLT in the control group (b)	(1). SLT (articulation, breathing, and relaxation, lip, and facial muscle training, 5 sessions a week for 4 weeks)	FDA	Significant differences in effective rates (*P* < 0.05)	None	6	Local	+	Strong	Superficial	+
Lai (2011)	(a) 65 (44/22) (b) 63 (45/18)	(a) 61.8 (b) 59.9	(a) 114 (b) 105	(1). AT (GV20, CV23, and two para-CV23 points, bleeding AT of the EX-HN12 and EX-HN13; 5 sessions a week for 4 weeks, 30 min. per session). (2). The same regime using SLT in the control group (b)	(1). SLT (articulation, breathing, and relaxation, lip, and facial muscle training, 5 sessions a week for 4 weeks)	FDA	Significant differences in effective rates (*P* < 0.05)	None	6	Local	+	Strong	Superficial	—
Luo (2012)	(a) 32 (18/14) (b) 28 (17/11)	(a) 56.2 (b) 57.4	(a) 72.1 (b) 71.3	(1). AT (CV23 and two para-CV23 points, Kai Yin, GB20, GB12, GV15, BL10; a total of 30 sessions, 40 min. per session). (2). The same regime using SLT in the control group (b)	(1). SLT (passive tongue and lip movement (severe), active tongue and lip movement training, articulation, and speech speed training (moderate), a total of 30 times)	SFG	Significant differences in effective rates (*P* < 0.05)	Pain on the AT-treated site (*n* = 1)	11	Local	+	Strong	Deep	—

Shi (2012)	(a) 30 (18/12) (b) 27 (16/11)	(a) 60.1 (b) 59.8	(a) 7.8 (b) 6.9	(1). AT (CV21, left and right EX-HN1 points, GB20, TE17, EX-HN10, CV23, bleeding AT of the EX-HN12 and EX-HN13; a total of 2 weeks, 30 min. per session). (2). The same regime using SLT in the control group (b)	(1). SLT (articulation, breathing, and relaxation, lip, and facial muscle training; a total of 2 weeks)	SFG	Significant differences in effective rates (*P* < 0.05)	None	11	Local	+	Strong	Deep	—
Liu (2012)	(a) 30 (18/12) (b) 30 (19/11)	(a) 56.0 (b) 55.4	(a) 71 (b) 72	(1). AT (GV26, PC6, HT5, LI4, GB20, GV15, CV23, Para-CV23, Upper-CV23, Han Xia, bleeding AT of the EX-HN12 and EX-HN13; 5 sessions a week for 90 days, 30 min. per session). (2). The same regime using SLT in the control group (b)	(1). SLT (articulation, breathing, and relaxation, lip, and facial muscle training; a total of 90 days, 30 min. per session)	SFG	Significant differences in effective rates (*P* < 0.05)	None	18	Broad	+	Strong	Deep	+

Liang (2014)	(a) 65 (b) 65	52.29	3–7	(1). AT (GV20, CV23, and two para-CV23 points, bleeding AT of the EX-HN12 and EX-HN13; 5 sessions a week for 4 weeks, 30 min. per session). (2). The same regime using SLT in the control group (b)	(1). SLT including articulation, breathing, and relaxation, lip, and facial muscle training, 5 sessions a week for 4 weeks	FDA	Significant differences in effective rates (*P* < 0.05)	None	6	Local	+	Strong	Superficial	+
Han (2014)	(a) 30 (20/10) (b) 30 (18/12)	(a) 58.2 (b) 56.4	(a) 28 (b) 29	(1). AT (GB20 and two points 4 cm under the GB 20, EX-HN14, Zhi Qiang, Tun Yan, Fa Yin, EX-HN 10, bleeding AT of the EX-HN12 and EX-HN13; a total of 40 days, 30 min. per session). (2). The same regime using SLT in the control group (b)	(1). SLT (articulation, breathing, and relaxation, lip, and facial muscle training; a total of 40 days, 30 min. per session)	FDA	Significant differences in effective rates and the numbers of FDA-a values (*P* < 0.05)	Pain on the AT-treated site (*n* = 1)	13	Local	+	Weak	Superficial	—

Feng (2015)	(a) 72; (b) 72	(a) >18, (b) >18	Undocumented	(1). AT (TE17, GB20, GB12, GV15, CV 23, and two para-CV23 points, CV22, PC6; daily, a total of 20 sessions, 30 min. per session)	General management	FDA	Significant differences in the total scores and effective rates (*P* < 0.01)	None	13	Broad	—	Strong	Deep	+
Li (2015)	(a) 89; (b) 85	60.34	3.9	(1). AT (GV20, CV23, and two para-CV23 points, bleeding AT of the EX-HN12 and EX-HN13; 5 sessions a week for 4 weeks, 30 min. per session). (2). The same regime using SLT in the control group (b)	(1). SLT (articulation, breathing, and relaxation, lip, and facial muscle training; five sessions a week for four weeks, 20 min. per session)	FDA	Significant differences in effective rates (*P* < 0.05)	None	6	Local	+	Strong	Superficial	+
Wang (2016)	(a) 60; (b) 60	42–79	Undocumented	(1). AT (GV20, CV23, and two para-CV23 points, bleeding AT of the EX-HN12 and EX-HN13, 5 sessions a week for 4 weeks, 30 min. per session). (2). The same regime using SLT in the control group (b)	(1). SLT (articulation, breathing, and relaxation, lip, and facial muscle training, 5 sessions a week for 4 weeks, 20 min. per session)	SFG	Significant differences in effective rates (*P* < 0.05)	None	6	Local	+	Strong	Superficial	+
Guan (2016)	(a) 60 (36/24) (b) 60 (40/20)	(a) 60.36 (b) 61.57	6	(1). AT (CV23 and two para-CV23 points, GB20, Gong Xue, Ex-HN14, GB19; daily, a total of 20 sessions, 20 min. per session)	General management	FDA	Significant differences in effective rates (*P* < 0.05)	None	11	Local	—	Weak	Deep	—
Jia (2016)	(a) 30 (18/12) (b) 30 (18/12)	(a) 62.3 (b) 62.4	Undocumented	(1). Tongue AT (division into two of the entire tongue substance, based on the midline. After that, vertical division into three and finally determining six regions of the tongue; left-upper, right-upper, left-middle, right-middle, left-lower, and right-lower region. Quick pricking with bleeding, a total of 12 punctures on each region. 5 sessions a week for 4 weeks). (2). The same regime using SLT in the control group (b)	(1). SLT (articulation, breathing, and relaxation, lip, and facial muscle training; a total of 90 days, 30 min. per session)	FDA	Significant differences in effective rates (*P* < 0.05)	None	6	Local	+	Strong	Superficial	—

Yang (2017)	(a) 40 (26/14) (b) 40 (27/13)	(a) 57.7 (b) 57.0	(a) 73 (b) 73	(1). Scalp EA (acupuncturing of 1 needle from the top of the ear to the GV20, vertically. Addition of two needling, anterior and posterior to the 2.5 cm position of the first acupoint; 5 sessions a week for 4 weeks, 30 min. per session). (2). The same regime using SLT in the control group (b)	(1). SLT (articulation, breathing, and relaxation, lip, and facial muscle training; a total of 4 weeks, 30 min. per session)	FDA	Significant differences in effective rates (*P* < 0.05)	None	6	Local	—	Strong	Superficial	—
Kang (2017)	(a) 44 (23/21) (b) 44 (24/20)	(a) 53.6 (b) 53.9	(a) 6 (b) 5	(1). AT (GV20, CV23, and two para-CV23 points, bleeding AT of the EX-HN12 and EX-HN13, 5 sessions a week for 40 days, 30 min. per session). (2). The same regime using SLT in the control group (b)	(1). SLT (articulation, breathing, and relaxation, lip, and facial muscle training; a total of 40 days, 30 min. per session)	FDA	Significant differences in effective rates and the numbers of FDA-a values (*P* < 0.05)	None	6	Local	+	Strong	Superficial	+

Acupoint location: the two para-CV23 points are located 2.5 cm from the left and right sides of the CV23; the upper-CV23 point is located 2.5 cm over the CV23; the Kai Yin points are located 1.5 cm under the left and right mandibular angles; the Gong Xue points are located 4 cm under both GB 20 points; Zhi Qiang is located on the midline between the hyoid bone and the upper margin of the thyroid cartilage; Tun Yan is located 1.3 cm from the left and right sides of the midline point between the hyoid bone and Adam's apple; Fa Yin points are located 0.5 cm from the left and right sides of the midline point between the thyroid and cricoid cartilages. In Shi's study, the two left and right Ex-HN1 points were selected among the four EX-HN1 points; the Han Xia is located at the middle point of the line which goes from the mandibular angle to the midpoint of the lower jaw. AT: acupuncture treatment, FDA: Frenchay dysarthria assessment, SFG: speech function grading, SLT: speech-language therapy, Total no: total numbers of needles.

**Table 2:** Risk of bias of the studies included in the review.

Category	Luo (2012)	Feng (2015)	Liang (2014)	Wang (2016)	Li (2015)	Guan (2016)	Zeng (2011)	Hu (2011)	Lai (2011)	Shi (2012)	Han (201 )	Liu (2012)	Jia (2016)	Yang (2017)	Kang (2017)
(1). Was the method of randomization adequate?	H	L	H	H	L	H	H	H	L	L	L	H	H	H	H
(2). Was the treatment allocation concealed?	U	U	U	U	U	U	U	U	L	U	U	L	U	U	U
(3). Was the patient blinded to the intervention?	U	U	U	U	U	U	U	U	U	U	U	U	U	U	U
(4). Were the personnel blinded to the intervention?	U	U	U	U	U	U	U	U	U	U	U	U	U	U	U
(5). Was the outcome assessor blinded to the intervention?	U	U	U	U	U	U	U	U	L	U	U	L	U	U	U
(6). Were incomplete outcome data addressed?	U	U	U	U	L	U	U	L	L	L	U	L	L	U	U
(7). Are reports of the study free of suggestion of selective outcome reporting?	L	L	L	L	L	L	L	L	L	L	L	L	L	L	L
(8). Was the study free of other problems that could put it at a high risk of bias?	U	U	U	U	U	U	U	U	U	U	U	U	U	U	U

H: high risk, L: low risk, U: uncertain.

**Table 3:** Meta-ANOVA results of acupuncture treatments for dysarthria according to manipulation types.

Manipulation type	Number of groups	Effect size and 95% CI			Test for heterogeneity		Test for subgroup differences	
		Risk ratio	Lower limit	Upper limit	*Q*-value	*I* ^2^ (%)	*Q*-value (DoF)	*P* value
Number of needles	Large (*n* = 7)	1.318	1.169	1.485	11.39	47.3	0.29 (1)	0.592
	Small (*n* = 8)	1.371	1.261	1.490	9.230	24.1		
Distribution of needles	Local (*n* = 12)	1.350	1.247	1.463	18.32	40.0	0.03 (1)	0.867
	Broad (*n* = 3)	1.328	1.108	1.593	3.890	48.6		
Bleeding of sublingual veins	Bleeding (*n* = 12)	1.380	1.281	1.488	14.38	23.5	2.02 (1)	0.155
	Nonbleeding (*n* = 3)	1.233	1.075	1.413	3.69	45.8		
Intensity of manipulation	Strong (*n* = 13)	1.341	1.257	1.431	14.40	16.7	0.17 (1)	0.681
	Weak (*n* = 2)	1.509	0.863	2.641	7.39	86.5		
Depth of needling	Deep (*n* = 6)	1.239	1.142	1.325	4.95	0.0	3.74 (1)	0.053
	Superficial (*n* = 9)	1.402	1.276	1.540	13.47	40.6		
Repetition of manipulation	Repetition (*n* = 8)	1.355	1.240	1.482	11.44	38.8	0.04 (1)	0.835
	Nonrepetition (*n* = 7)	1.334	1.180	1.508	10.73	44.1		

CI: confidence interval, DoF: degrees of freedom.

## Data Availability

Data used to support the findings of this study are included within the supplementary information files.

## Supplementary Materials

Supplementary Materials Supplementary file 1 lists the PRISMA checklist of this study. Supplementary file 2 summarizes the intervention types, improved cases in the treatment group and the control group, and mean and standard deviation values of the FDA total scores and the FDA-*a*-points between pre- and post-treatment periods.
